# The Disposition of the Appendiceal Stump

**Published:** 1907-11

**Authors:** Benjamin Merrill Rickets

**Affiliations:** Cincinnati, Ohio


### THE DISPOSITION OF THE APPENDICEAL STUMP.*

By BENJAMIN MERRILL RICKETS, Ph.B., M.D., LL.D.,

Cincinnati, Ohio.

---

This subject was dealt with from two view-points. First—The consideration of the various methods of disposing of the stump. Second—The cause of hemorrhage and its frequency.

In response to circular letters sent to a number of surgeons, sixty-four replies were received, representing 80,251 appendectomies, with an average mortality of seven and one-half in acute and one and three-quarters per cent. in chronic cases. Eight per cent. of the total number of operations reported have been

(*Author's abstract read before the Mississippi Valley Med. Asso., Columbus, O., Oct. 8-10 1907.)

done by the four purse-string methods. The number of acute and chronic cases was about equal.

The various per cents of different techniques used are given. Also the per cent. of cases in which the different ligatures and sutures of catgut and linen were used.

About sixty cases of hemorrhages from the stump are mentioned, forty of which are tabulated in detail, giving eleven deaths. Of the total number of hemorrhages mentioned, all but one have resulted in the purse-string methods. The technique of each of the sixty-four surgeons reporting is given briefly. Investigation of this subject shows that many operators have abandoned the purse-string methods; that others are doing so; that simple ligature for the firm and extirpation for the soft appendix are the safer methods and that any other than simple ligature and extirpation with linen or silk for ligature or suture should be condemned absolutely.

---

Dr. Samuel G. Dixon, Pennsylvania State Commissioner of Health, has ordered that the sheets on beds in Pullman sleepers be made longer so as to prevent the blankets from coming in contact with the faces of the occupants. He also issued an order forbidding porters to brush clothing in the aisles of the cars.

---

Germany and Russia have signed a special convention allowing each country to install special sanitary officials in the border towns in case the present epidemic of cholera should spread to them. A St. Petersburg exchange states that the epidemic in Russia is spreading with giant strides, especially along the Volga. About 2,000 cases had been officially recorded up to September 7. At Bialystok a penalty of 3,000 roubles is to be enforced in case of neglect of the sanitary regulation.

---

Baltimore has a genuine epidemic of typhoid fever. The incidence of the disease in physicians and politicians is unusual. So far as the individuals attacked are concerned this distinction is probably unmerited, but if physicians and politicians should suffer a little more than their indicated share of the disease the discrimination would not be unjust and might prove salutary. The city is not at all warmed up to the situation. Of course, the epidemic will pass; it is now passing. All conditions are normal except the figures.—Maryland Med. Jour.

